# Structured reporting of computed tomography in the staging of colon cancer: a Delphi consensus proposal

**DOI:** 10.1007/s11547-021-01418-9

**Published:** 2021-11-06

**Authors:** Vincenza Granata, Lorenzo Faggioni, Roberta Grassi, Roberta Fusco, Alfonso Reginelli, Daniela Rega, Nicola Maggialetti, Duccio Buccicardi, Barbara Frittoli, Marco Rengo, Chandra Bortolotto, Roberto Prost, Giorgia Viola Lacasella, Marco Montella, Eleonora Ciaghi, Francesco Bellifemine, Federica De Muzio, Giulia Grazzini, Massimo De Filippo, Salvatore Cappabianca, Andrea Laghi, Roberto Grassi, Luca Brunese, Emanuele Neri, Vittorio Miele, Francesca Coppola

**Affiliations:** 1 Division of Radiology, “Istituto Nazionale Tumori IRCCS Fondazione Pascale – IRCCS di Napoli”, Naples, Italy; 2 Department of Translational Research, University of Pisa, Pisa, Italy; 3 Italian Society of Medical and Interventional Radiology (SIRM), SIRM Foundation, via della Signora 2, 20122 Milan, Italy; 4 Division of Radiology, “Università Degli Studi Della Campania Luigi Vanvitelli”, Naples, Italy; 5 Medical Oncology Division, Igea SpA, Naples, Italy; 6 Division of Colorectal Surgery, Istituto Nazionale Tumori IRCCS Fondazione Pascale-IRCCS Di Napoli, 80131 Naples, Italy; 7 Section of Radiodiagnostic, DSMBNOS, “Aldo Moro” University, Bari, Italy; 8 Imaging Department, San Paolo Hospital, Savona, Italy; 9 Department of Radiology, Spedali Civili Hospital of Brescia, University of Brescia, Brescia, Italy; 10 Department of Radiological Sciences, Oncology and Pathology, Sapienza University of Rome - I.C.O.T. Hospital, Via Franco Faggiana, 1668, 04100 Latina, Italy; 11 Department of Radiology, I.R.C.C.S. Policlinico San Matteo Foundation, Pavia, Italy; 12 Radiology Unit, Azienda Ospedaliera Brotzu, Cagliari, Italy; 13 Exprivia Spa, Bari, Italy; 14 Department of Medicine and Health Sciences “V. Tiberio”, University of Molise, Via Francesco De Sanctis 1, 86100 Campobasso, Italy; 15 Division of Radiology, “Azienda Ospedaliera Universitaria Careggi”, Florence, Italy; 16 Department of Medicine and Surgery, Unit of Radiologic Science, University of Parma, Maggiore Hospital, Parma, Italy; 17 Department of Surgical and Medical Sciences and Translational Medicine, Sapienza University of Rome-Sant’Andrea University Hospital, Via di Grottarossa, 1035-1039, 00189 Rome, Italy; 18 Department of Radiology, IRCCS Azienda Ospedaliero-Universitaria Di Bologna, Bologna, Italy

**Keywords:** Radiology report, Structured report, Colon cancer, Computed tomography

## Abstract

**Background:**

Structured reporting (SR) in radiology is becoming increasingly necessary and has been recognized recently by major scientific societies. This study aims to build structured CT-based reports in colon cancer during the staging phase in order to improve communication between the radiologist, members of multidisciplinary teams and patients.

**Materials and methods:**

A panel of expert radiologists, members of the Italian Society of Medical and Interventional Radiology, was established. A modified Delphi process was used to develop the SR and to assess a level of agreement for all report sections. Cronbach’s alpha (Cα) correlation coefficient was used to assess internal consistency for each section and to measure quality analysis according to the average inter-item correlation.

**Results:**

The final SR version was built by including *n* = 18 items in the “Patient Clinical Data” section, *n* = 7 items in the “Clinical Evaluation” section, *n* = 9 items in the “Imaging Protocol” section and *n* = 29 items in the “Report” section. Overall, 63 items were included in the final version of the SR. Both in the first and second round, all sections received a higher than good rating: a mean value of 4.6 and range 3.6–4.9 in the first round; a mean value of 5.0 and range 4.9–5 in the second round. In the first round, Cronbach’s alpha (Cα) correlation coefficient was a questionable 0.61. In the first round, the overall mean score of the experts and the sum of scores for the structured report were 4.6 (range 1–5) and 1111 (mean value 74.07, STD 4.85), respectively. In the second round, Cronbach’s alpha (Cα) correlation coefficient was an acceptable 0.70. In the second round, the overall mean score of the experts and the sum of score for structured report were 4.9 (range 4–5) and 1108 (mean value 79.14, STD 1.83), respectively. The overall mean score obtained by the experts in the second round was higher than the overall mean score of the first round, with a lower standard deviation value to underline greater agreement among the experts for the structured report reached in this round.

**Conclusions:**

A wide implementation of SR is of critical importance in order to offer referring physicians and patients optimum quality of service and to provide researchers with the best quality data in the context of big data exploitation of available clinical data. Implementation is a complex procedure, requiring mature technology to successfully address the multiple challenges of user-friendliness, organization and interoperability.

**Supplementary Information:**

The online version contains supplementary material available at 10.1007/s11547-021-01418-9.

## Introduction

The clear communication of imaging features to referring physicians is critical for patient management. The data contained move both the decision-making procedure and subsequent treatment. Radiologists’ reports still represent the gold standard concerning comprehensiveness and accuracy [1, 2]. Radiology reports are habitually produced as non-structured free text (FRT). However, variations with regard to content, style and presentation can hamper information transfer and reduce the precision of the reports, which can in turn adversely affect the extraction of the required key information by the referring physician [2]. At worst, the consequential communication errors can lead to improper diagnosis, deferred initiation of satisfactory therapy, with adverse patient outcome. The American Recovery and Reinvestment Act and the Health Information Technology for Economic and Clinical Health specified that structuring data in health records lead to a significant progress in outcome of patients [1, 2]. Since the radiology report is part of the health record, FRT should be systematized and shifted toward structured report (SR). The query of whether all diagnostic processes should have SRs is open [2, 3]. The main purposes for a shift from FTR to SR focus on three key features: quality, datafication/quantification and accessibility [2]. The use of templates provides a checklist as to whether all relevant items for a specific technique are addressed. Moreover, thanks to this “structure,” the radiological report allows for the association of radiological data and other key clinical features, leading to a precise diagnosis and personalized medicine. With regard to accessibility, it is well known that radiology reports are a rich source of data for research. This allows automated data mining, which may help to validate the relevance of imaging biomarkers by highlighting the clinical contexts in which they are most appropriate and helping to devise potential new application domains. For this reason, radiology reports should be structured with content based on standard terminology and should be accessible via standard access mechanisms and protocols [2].

Despite the obvious advantages, structured templates have not yet become established in the radiological routine. The reasons for this include the current lack of usable templates and the minimal availability of software solutions for SR [4]

In this scenario, the Italian Society of Medical and Interventional Radiology (SIRM) created an Italian warehouse of SR templates that can be freely accessed by all SIRM members, with the purpose of its routine use in a clinical setting [3, 5, 6].

The role of imaging in clinically staging colorectal cancer has grown substantially in the twenty-first century, with more widespread availability of multi-row detector computed tomography (CT) [7–9], high-resolution magnetic resonance imaging (MRI) [10–15] and integrated positron-emission tomography (PET)/CT [16, 17]. In contrast to staging many other cancers, increasing the colorectal cancer stage is not highly correlated with survival. For locally advanced, amenable to resection, non-metastatic colon cancer (T1N0 to T4bN4), colectomy or endoscopic surgery followed by chemotherapy is recommended, and for those amenable to resection, metastatic colon cancer (CC) neoadjuvant chemotherapy and colectomy with/without chemotherapy are recommended [18]. The 5-year survival rates of patients with stage I, II and III CC are ∼ 93, 80 and 60%, respectively [19]. The American Joint Committee on Cancer (AJCC) TNM staging system is widely used to assess the prognosis of patients with CC [20]. However, the prognosis of patients with CC at the same stage varies widely, and the accuracy of TMN staging as a predictive approach has certain limitations [21]. Therefore, another approach is needed to identify patients with poor prognosis to allow for the development of individualized treatment and monitoring approaches [21].

The correct stratification of patients requires an accurate diagnosis not only of the TNM, but also the identification of various risk factors (e.g., mucinous tumor), as well as adequate communication between the radiologist and multidisciplinary group, communication that takes place via radiological reports. To improve this communication and meet the needs of clinicians, the aim of this study is to propose a SR template for colon cancer based on CT to guide radiologists in the systematic reporting of neoplasm findings at the staging phase.

## Materials and methods

### Panel expert

Following extensive discussion between expert radiologists, a multi-round consensus-building Delphi exercise was performed to develop a comprehensive focused SR template for CT at the staging phase of patients with colon cancer.

A SIRM radiologist, expert in abdominal imaging, created the first draft of the SR for CT colon cancer with the collaboration of a surgeon specializing in colon cancer.

A working team of 15 experts was set up, with members from the Italian College of Gastro-enteric Radiologists and of Diagnostic Imaging in Oncology Radiologists from SIRM. Their aim was to revise the initial draft iteratively, with the objective of reaching a final consensus on SR.

### Selection of the Delphi domains and items

All the experts reviewed literature data on the main scientific databases, including PubMed, Scopus and Google Scholar, to assess papers on colon cancer CT and structured radiology reports from December 2000 to May 2021. The full text of the selected studies was reviewed by all members of the expert panel, and each of them developed and shared the list of Delphi items via emails and/or teleconferences.

The SR was divided into four sections: (a) Patient Clinical Data, (b) Clinical Evaluation, (c) Imaging Protocol and (d) Report. A dedicated section of significant images was added as part of the report.

Two Delphi rounds were performed. During the first round, each panelist independently contributed to refining the SR draft by means of online meetings or email exchanges. The level of panelists’ agreement for each SR model was tested in the second Delphi through a Google Form questionnaire shared by email. Each expert expressed individual comments for each specific template section by using a five-point Likert scale (1 = strongly disagree, 2 = slightly disagree, 3 = slightly agree; 4 = generally agree, 5 = strongly agree).

After the second Delphi round, the last version of the SR was generated on the dedicated RSNA website (radreport.org) by using a T-Rex template format, in line with IHE (Integrating Healthcare Enterprise) and the MRRT (management of radiology report templates) profiles, accessible as open-source software, with the technical support of Exprivia (Exprivia SpA, Bari, Italy). These determine both the format of radiology report templates [using version 5 of Hypertext Markup Language (HTML5)] and the transporting mechanism to request, retrieve and stock these schedules [22]. The radiology report was structured by using a series of “codified queries” integrated in the T-Rex editor’s preselected sections [22].

### Statistical analysis

Answers from each panelist were exported to a Microsoft Excel® format for ease of data collection and statistical analysis.

All ratings of panelists for each section were analyzed with descriptive statistics measuring the mean score, the standard deviation and the sum of scores. A mean score of 3 was considered good and a score of 4 excellent.

To measure the internal consistency of the panelist ratings for each section of the report, a quality analysis based on the average inter-item correlation was performed with Cronbach’s alpha (Cα) correlation coefficient [23, 24]. The Cα test provides a measure of the internal consistency of a test or scale; it is expressed as a number between 0 and 1. Internal consistency describes the extent to which all the items in a test measure the same concept. Cα was determined after each round.

The closer Cα coefficient is to 1.0, the greater the internal consistency of the items in the scale. An alpha coefficient of (*α*) ≥ 0.9 was considered excellent, *α* ≥ 0.8 good, *α* ≥ 0.7 acceptable, *α* ≥ 0.6 questionable, *α* ≥ 0.5 poor and *α* < 0.5 unacceptable. However, during the iterations an *α* of 0.8 was considered a reasonable goal for internal reliability.

The data analysis was performed using MATLAB’s Statistic Toolbox (The MathWorks, Inc., Natick, MA, USA).

## Results

### Structured report

The final SR (Appendix 1 in Electronic of Supplementary Materials) version was built by including *n* = 18 items in the “Patient Clinical Data” section, *n* = 7 items in the “Clinical Evaluation” section, *n* = 9 items in the “Imaging Protocol” section and *n* = 29 items in the “Report” section. Overall, 63 items were included in the final version of the SR.

The “Patient Clinical Data” section included patient clinical data, previous or family history of malignancies, risk factors or predisposing pathologies. In this section, we included the item “Allergies” to drug or no drug and contrast medium.

The “Clinical Evaluation” section collected previous examination results, a genetic panel and clinical symptoms.

The “Imaging Protocol” section included data on the equipment used, the number of detectors and whether it was multidetector or dual energy, including data on the reconstruction algorithm and slice thickness. In addition, we collected data on contrast study protocol, including data on the contrast study phase, as well as data concerning the contrast medium, such as the active principle, commercial name, dosage, flow rate, concentration and ongoing adverse events.

The “Report” section included data on lesion site, type, size, presence of prosthesis and/or colostomy, local invasion, tumor stage, node stage and metastases stage, as well as the presence of peritoneal carcinomatosis, according to the peritoneal carcinomatosis index (PCI) described by Sugarbaker [25].

### Consensus agreement

Table 1 reports the single score and sum of scores the 15 panelists gave for the structured report in the first round. One of the experts did not participate in the second round, with a total of 14 panelists for this round. Table 2 reports the single score and sum of scores the panelists gave the structured report in the second round.

**Table 1:** Single score and sum of scores of the 15 panelists for structured report in the first round

Panelist #	A1. Anthropometric data	A2. Personal assessments	A3. Allergies and adverse reactions	B1. Clinical information	C1. Examination data	C2. Contrast agent	C3. Study protocol	C4. Adverse Events	D1.Lesion	D2. Lymphadenopathy	D3. Distant metastasis	D4. Carcinosis	D5. Free payment	D6. Perforation	D7. TNM stage	D8. Accessory findings	Tot
1	5	3	3	5	5	5	5	5	4	5	5	5	5	5	4	5	74
2	2	2	5	5	5	5	5	5	5	5	5	5	5	5	5	3	72
3	5	5	5	5	4	5	5	5	5	5	5	5	5	5	5	5	79
4	5	3	5	5	5	5	5	5	4	5	4	5	5	5	5	5	76
5	3	1	5	5	5	5	5	5	5	5	5	5	5	5	5	5	74
6	3	1	4	5	5	5	4	5	5	5	5	5	5	5	2	3	67
7	5	5	5	5	5	5	5	5	5	5	5	5	5	4	5	5	79
8	5	5	5	4	4	4	4	4	5	4	5	4	5	5	5	4	72
9	3	3	5	5	5	5	5	5	5	5	5	5	5	5	4	5	75
10	1	4	1	3	5	5	5	5	5	5	5	5	5	5	5	5	69
11	3	4	4	4	5	5	3	5	2	3	3	5	5	3	5	5	64
12	5	5	5	5	5	5	5	5	5	5	5	5	5	5	5	5	80
13	5	5	5	5	5	5	5	5	5	5	5	5	5	5	5	5	80
14	5	5	5	5	5	5	3	5	5	5	5	5	5	5	5	5	78
15	4	3	5	5	5	5	5	5	5	5	5	5	5	4	5	1	72
Mean	3.93	3.60	4.47	4.73	4.87	4.93	4.60	4.93	4.67	4.80	4.80	4.93	5.00	4.73	4.67	4.40	74.07
Std	1.33	1.45	1.13	0.59	0.35	0.26	0.74	0.26	0.82	0.56	0.56	0.26	0.00	0.59	0.82	1.18	4.85

**Table 2:** Single score and sum of scores of the 14 panelists for structured report in the second round

Panelist #	A1. Anthropometric data	A2. Personal assessments	A3. Allergies and adverse reactions	B1. Clinical information	C1. Examination data	C2. Contrast agent	C3. Study protocol	C4. Adverse Events	D1.Lesion	D2. Lymphadenopathy	D3. Distant metastasis	D4. Carcinosis	D5. Free payment	D6. Perforation	D7. TNM stage	D8. Accessory findings	Tot
1	5	5	5	5	5	5	5	5	5	5	5	5	5	5	5	5	80
2	5	5	5	5	5	5	5	5	5	5	5	5	5	5	5	5	80
3	5	5	5	5	5	5	5	5	5	5	5	5	5	5	5	5	80
4	5	5	5	5	5	5	5	5	5	5	5	5	5	5	5	5	80
5	5	5	5	4	5	5	5	5	5	4	4	5	5	5	5	5	77
6	5	3	5	5	5	5	5	5	5	5	5	5	4	5	5	5	77
7	5	5	5	5	5	5	5	5	5	5	5	5	5	5	5	5	80
8	5	5	5	5	5	5	5	5	5	5	5	5	5	5	5	5	80
9	5	5	5	5	5	5	5	5	5	5	5	5	5	5	5	5	80
10	5	5	5	5	5	5	5	5	5	5	5	5	5	5	5	5	80
11	4	3	4	5	5	5	5	5	5	5	5	5	5	5	5	3	74
12	5	5	5	5	5	5	5	5	5	5	5	5	5	5	5	5	80
13	5	5	5	5	5	5	5	5	5	5	5	5	5	5	5	5	80
14	5	5	5	5	5	5	5	5	5	5	5	5	5	5	5	5	80
Mean	4.93	4.71	4.93	4.93	5.00	5.00	5.00	5.00	5.00	4.93	4.93	5.00	4.93	5.00	5.00	4.86	79.14
STD	0.27	0.73	0.27	0.27	0.00	0.00	0.00	0.00	0.00	0.27	0.27	0.00	0.27	0.00	0.00	0.53	1.83

Both in the first and second round, as reported in Table 1 and 2, all sections received more than a good rating: a mean value of 4.6 and range 3.6–4.9 in the first round; a mean value of 5.0 and range 4.9–5 in the second round.

In the first round, Cronbach’s alpha (Cα) correlation coefficient was a questionable 0.61. In the first round, the overall mean score of the experts and the sum of scores for the structured report were 4.6 (range 1–5) and 1111 (mean value 74.07, STD 4.85), respectively.

In the second round, Cronbach’s alpha (Cα) correlation coefficient was an acceptable 0.70. In the second round, the overall mean score of the experts and the sum of scores for the structured report were 4.9 (range 4–5) and 1108 (mean value 79.14, STD 1.83), respectively.

The overall mean score of the experts in the second round was higher than the overall mean score of the first round, with a lower standard deviation value to underline greater agreement among the experts in the structured report reached in this round.

## Discussion

To the best of our knowledge, this was the first time that a group of experts promoted the creation of a structured report for the staging of colon cancer, based on a multi-round consensus-building Delphi exercise following in-depth discussion between expert radiologists in gastro-enteric and oncological imaging. In our previous study [3], we assessed a SR template for rectal cancer (RC) MRI during staging and re-staging phases. Although the RC MRI templates are based on a multi-round consensus-building Delphi exercise, in this study the draft of the SR proposed derives from the multidisciplinary agreement of a radiologist with a surgeon. A multidisciplinary validation of the SR is necessary, because precision medicine today is based on a multidisciplinary management of the patient.

The final SR version was divided into four sections: *(a)* Patient Clinical Data, *(b)* Clinical Evaluation, *(c)* Imaging Protocol and *(d)* Report, including 63 items. In both the first and second rounds, all sections received more than a good rating; however, the weakest sections were “Patient Clinical Data” and “Clinical Evaluation.” In the first round, Cronbach’s alpha (Cα) correlation coefficient was a questionable 0.61. In the second round, Cronbach’s alpha (Cα) correlation coefficient was an acceptable 0.70.

Weiss et al. described three levels of SR [26]: (a) The first level is a structured format with paragraphs and subheadings. Currently, almost all radiology reports contain this structure, with sections for clinical information, examination protocol, radiological findings and a conclusion to highlight the most important findings; (b) the second level refers to a consistent organization; and (c) the third level directly addresses the consistent use of dedicated terminology, namely standard language. The present SR is a third-level SR since it is based on standardized terminology and structures, features required to adhere to diagnostic–therapeutic recommendations and enrollment in clinical trials and to reduce any ambiguity that may arise from non-conventional lexicon. Thanks to this “structure,” this report may allow the association of radiological data and other clinical features in order to obtain personalized medicine. In fact, as regards accessibility, it is well known that radiology reports are a rich source of data for research. This allows for automated data mining, which may help to validate the relevance of imaging biomarkers by highlighting the clinical contexts in which they are most appropriate and useful in devising potential new application domains [27–35]. As a means of obtaining large databases (and considering the fact that only the “Report” section is mandatory, while the others do not have to be compiled, so as not to slow down workflow), in the second round good agreement was also reached in the “Patient Clinical Data” and “Clinical Evaluation” sections.

In the “Imaging Protocol” section, sharing the examination technique (not only within one's own department but also with the radiology departments of other centers) allows for the standardization and optimization of study protocols. For example, during follow-up, differences in acquisition parameters and segmentation algorithms are important features that can lead to variability in volumetric assessment. Thus, slice thickness and other protocol-related features such as the reconstruction kernel and field of view should remain constant for reliable measurements to be performed. In the protocol optimization stage, enhanced communication between different centers can theoretically lead to quality improvement through enhanced patient safety, contrast optimization and image quality [36–38].

A final consideration should be made on the possibility of using a template to guide the radiologist’s description of important radiological features that might be omitted in a free text report through simple distraction. For example, in our report a significant section is dedicated to the description of peritoneal carcinosis. A correct evaluation of this (thereby referring to Sugarbaker’s peritoneal carcinosis index [22]) allows for a stratification of patients and avoids unnecessary surgery. Using a checklist and a systematic search pattern may help to prevent such diagnostic errors. Both radiologists and referring clinicians are keen to reduce the rate of diagnostic errors, which for radiologists accounts for as much as 4% of reports [39–43]. A retrospective review of 3000 MRI examinations helped identify clinically significant extraspinal findings in 28.5% of patients which were not included in the original unstructured report [44]. Similarly, the use of a checklist-style SRs has been shown to improve the rate of diagnosis of non-fracture-related findings on cervical CT [45]. In addition, SRs have been shown to enhance clinical impact on tumor staging and surgical planning for pancreatic and rectal carcinoma [46–48]. Brook et al. compared the results of SR versus FRT reporting of CT findings for the staging and the assessment of resectability in pancreatic cancer patients [46]. They concluded that surgeons were more confident about tumor resectability using SR compared to FTR [46]. Sahni et al. showed that the use of an MRI SR improved rectal cancer staging when compared to the use of an FTR [48].

The present study has several limits. Firstly, the panelists were of the same nationality; the contribution of experts from multiple countries would allow for broader sharing and would increase the consistency of the SR. Secondly, this study was not aimed toward assessing the impact of the SR on the management of patients with colon cancer in clinical setting. The future objective is the clinical validation of this report.

## Conclusion

The present SR, based on a multi-round consensus-building Delphi exercise, uses standardized terminology and structures, in order to adhere to diagnostic/therapeutic recommendations and facilitate enrollment in clinical trials, to reduce any ambiguity that may arise from non-conventional lexicon and to enable better communication between radiologists and clinicians. A standardized approach with best practice guidelines can offer the foundation for quality assurance measures within institutions. SR improves the quality, clarity and reproducibility of reports across departments, cities and countries, in order to assist patient management, improve patient healthcare and facilitate research.

## Supplementary Information

Below is the link to the electronic supplementary material.

Supplementary file1 (DOCX 267 kb)
