# Supplementary material for: Structured reporting of computed tomography in the staging of colon cancer: a Delphi consensus proposal
Source: Radiol Med. 2021 Nov 6;127(1):21–9. doi: 10.1007/s11547-021-01418-9 (PMC8795004; doi:10.1007/s11547-021-01418-9)
Supplement: Supplementary file 1 — Supplementary file1 (DOCX 267 kb) [file 11547_2021_1418_MOESM1_ESM.docx]

**PATIENT’S CLINICAL DATA (*imported from RIS)**

| FIELD | DETAIL | ADMITTED VALUES | |
| --- | --- | --- | --- |
| ANTHROPOMETRIC DATA | | | |
| Weight |  | (kg) *[Numeric]* | |
| Height |  | (cm) *[Numeric]* | |
| BMI |  | *[Numeric]* (automatically calculated) | |
| BSA |  | *[Numeric]* (automatically calculated) | |
| Age |  | (years) *[Numeric]* | |
| Age range |  | - < 50 years - > 50 years | |
| PATIENT HISTORY | | | |
| Family history of colorectal cancer  (visible only if “Yes” and repeatable) | Yes/No |  | |
|  | Degree of kinship | - Mother - Father - Brother(s)/sister(s) - Maternal grandparent(s) - Paternal grandparent(s) - Uncle(s)/aunt(s) - Other *[free text]* | |
|  | Notes | *[free text]* | |
| Family history of cancer  (visible only if “Yes” and repeatable) | Yes/No |  | |
|  | Degree of kinship | - Mother - Father - Brother(s)/sister(s) - Maternal grandparent(s) - Paternal grandparent(s) - Uncle(s)/aunt(s) - Other *[free text]* | |
|  | Notes | *[free text]* | |
| Personal history of other cancers | Yes/No |  | |
|  | Notes | *[free text]* | |
| Predisposing diseases  (visible only if “Yes” and repeatable) | Yes/No |  | |
|  | Type | - Diabetes - Hypercholesterolemia - Arterial hypertension - Hypertriglyceridemia - Crohn disease - Ulcerative rectocolitis - Metabolic syndrome | |
|  | Notes | *[free text]* | |
| Hereditary genetic mutations  (visible only if “Yes” and repeatable) | Type | - Polyposis associated with MutYH or MAP mutation - Attenuated familial adenomatous polyposis (AFAP) - Classic adenomatous familial polyposis (FAP) - Lynch syndrome | |
| Other genetic mutations | Yes/No |  | |
|  | Notes | *[free text]* | |
| Risk factors  (visible only if “Yes” and repeatable) | Smoker | Yes/No | |
|  | SMOKING DETAILS (visible only if Smoke = “Yes”) | | |
|  |  | Smoker | - Current smoker - Former smoker |
|  |  | Cigarette smoke | Yes/No |
|  |  | Number of daily cigarettes (if “Current smoker”) | - Light (< 15) - Heavy (≥15) |
|  |  | Years of smoking | *[Numeric]* |
|  |  | Years from cessation  (if “Former smoker”) | - ≤15 - >15 |
|  |  | Cigarettes per year  (packs/year)  (if “Former smoker” or “Current smoker”) | *[Numeric]* (automatically calculated)*  *(Number of daily cigarettes x smoking years / 20) |
|  |  | Vaping | Yes/No |
|  |  | Number of daily electronic cigarette refills (if vaping = “Yes”) | *[Numeric]* |
|  |  | Number of years  (if vaping = “Yes”) | *[Numeric]* |
|  |  | Notes | *[free text]* |
|  | High alcohol intake | Yes* *(more than 1 glass/day for women and 2 glasses/day for men)  No | |
|  | High meat intake | Yes* *(white or red meat intake more than 3 times/week)  No | |
|  | High cured meat intake | Yes *(cured meat intake more than once a week)  No | |
|  | Low vegetable intake | Yes *(less than 2 servings/day)  No | |
|  | Low fruit intake | Yes (*less than 3 whole fruits/day)  No | |
|  | Notes | *[free text]* | |
| Microsatellite instability | Yes/No |  | |
|  | Notes | *[free text]* | |
| ALLERGIES AND ADVERSE REACTIONS | | | |
| Reported allergies  (visible only if “Yes” and repeatable) | Yes/No |  | |
|  | Type | - Drug-related (n of drugs) - Contrast medium-related (n of contrast media) - Drug-unrelated | |
|  | Active principle/molecule  [if drug- or contrast medium-related allergy] | *[free text]* | |
|  | Commercial name  [if drug- or contrast medium-related allergy] | *[free text]* | |
|  | Notes | *[free text]* | |
| PREVIOUS adverse reactions  (visible only if “Yes” and repeatable) | Yes/No |  | |
|  | Date | month/year [mm/yyyy] | |
|  | Type | Contrast medium-related / unrelated | |
|  | Degree | - Mild - Moderate - Severe | |
|  | Time of onset | - Early - Late | |
|  | Notes | *[free text]* | |
| Antiallergic premedication | Yes/No |  | |
|  | Treatment | - Steroid - Antihistamine | |
|  | Complete | Yes/No | |
|  | Notes | *[free text]* | |
| Nephroprotective protocol | Yes/No |  | |
|  | Complete | Yes/No | |
|  | Serum creatinine | *[Numeric]* (mg/dl) | |
|  | GFR (Glomerular Filtration Rate) | *[Numeric]* (ml/min)  <https://www.merckmanuals.com/medical-calculators/GFR_CKD_EPI-it.htm> (sex, race, age, serum creatinine) | |
|  | Notes | *[free text]* | |

**CLINICAL EVALUATION**

| FIELD | DETAIL | ADMITTED VALUES |
| --- | --- | --- |
| CLINICAL INFORMATION | | |
| Prior examinations  (visible only if “Yes” and repeatable) | Yes/No |  |
|  | Type | - Optical colonoscopy - Virtual colonoscopy - CT - MRI - US - PET/CT - Other *[free text]* |
|  | Date | *[dd/mm/yyyy]* |
|  | Notes | *[free text]* |
| Histopathological examination on biopsy specimen | Yes/No |  |
|  | Notes | *[free text]* |
| CEA level |  | *[Numeric]* |
| Blood count |  | *[Numeric]* |
| Serum creatinine |  | *[Numeric]* |
| Liver function |  | - Normal - Impaired |
| Symptoms |  | - Weight loss - Anemia - Altered bowel habits - Abdominal pain - Other *[free text]* |

**IMAGING PROTOCOL**

| FIELD | DETAIL | ADMITTED VALUES |
| --- | --- | --- |
| IMAGING DATA | | |
| Date of examination |  | Date *[dd/mm/yyyy]* |
| Clinical indication | Primary staging |  |
| Scanner brand and model |  | *[free text]* |
| Scanning technique | Number of detector rows | *[Numeric]* |
|  | Precontrast scan  ***(details visible only if “Yes”)*** | Yes/No  Dual energy (Yes/No)  Slice thickness (mm) *[Numeric]*  Convolution kernel(s) *[free text]*  Body area *[multiple choice]*:   - abdomen - chest - brain |
|  | Post-contrast scan  ***(details repeatable for each post-contrast scan)*** | *[Numeric]*  Post-contrast phase(s) (arterial, venous, late)  Dual energy (Yes/No)  Slice thickness (mm) *[Numeric]*  Convolution kernel(s) *[free text]*  Body area *[multiple choice]*:   - abdomen - chest - brain |
| Radiation exposure | Class of radiation exposure | *[Numeric]* |
| CONTRAST MEDIUM | | |
| Use of contrast medium  (visible only if “Yes”) | Yes/No |  |
|  | Active principle | - Iobitridol - Iodixanol - Iohexol - Iomeprol - Iopromide - Ioversol |
|  | Commercial name | *[free text]* |
|  | Volume | *[Numeric]* (ml) |
|  | Flow rate | *[Numeric]* (ml/sec) |
|  | Concentration | *[Numeric]* (mg I/ml) |
|  | Notes | *[free text]* |
| ADVERSE EVENTS | | |
| ONGOING adverse events  (visible only if “Yes”) | Yes/No |  |
|  | Date and hour of event | *[dd/mm/yyyy, hour]* |
|  | Degree | - Mild - Moderate - Severe |
|  | Time of onset | - Early - Late   Minutes *[Numeric]* (optional) |
|  | Type | ALLERGIC / ALLERGIC-LIKE  **Mild**   - Sparse wheals/itch - Skin oedema - Mild itching / feeling like ”velvet in the throat“ - Nasal congestion - Sneezing - Conjunctivitis - Rhinorrhoea   **Moderate**   - Diffuse wheals/intense itch - Diffuse skin oedema - Facial oedema without dyspnea - Feeling of choking or hoarseness - Wheezing / mild bronchospasm without hypoxia   **Severe**   - Dyspnea - Erythema – diffuse mucocutaneous symptoms - Laryngeal oedema with stridor and/or hypoxia - Wheezing / bronchospasm - Significant hypoxia - Anaphylactic shock (severe hypotension and brady-tachyarrhythmia)   NON-ALLERGIC  **Mild**   - Mild nausea/limited vomiting - Transient chills / heat / redness - Headache / dizziness / anxiety / altered taste - Slight increase in blood pressure - Self-limiting vasovagal reaction   **Moderate**   - Prolonged nausea/vomiting - Elevated arterial blood pressure - Isolated chest pain - Vasovagal reaction   **Severe**   - Treatment-refractory vasovagal reaction - Arrhythmia - Convulsions - Severe arterial hypertension   CONTRAST MEDIUM EXTRAVASATION |
|  | Type of treatment | - Wait and see - Drug therapy (specify in “Notes” field) - Anaesthesiologist’s intervention required |
|  | Event resolution | - Spontaneous - After treatment - After hospitalisation - Other [free text] |
|  | Notes | *[free text]* |

**REPORT**

| FIELD | DETAIL | | ADMITTED VALUES |
| --- | --- | --- | --- |
| DIAGNOSIS | | | |
| PRIMARY TUMOR | | | |
|  | | Lesion | - Visible - Not visible *(i.e., lesions endoscopically resected and subsequently characterized as cancer polyps)* |
|  |  | Type | - Vegetating - Infiltrating - Vegeto-infiltrating - Stenosing - Mucin component - Necrotic |
|  |  | Prosthesis | Yes/No  If yes:   - Correctly positioned - Migrated - Portion of endoprosthetic lesion (mm)  1. Obstructing 2. Non-obstructing |
|  |  | Colostomy | Yes/No |
|  |  | Site | - Caecum - Ascending colon - Hepatic flexure - Proximal transverse colon - Distal transverse colon - Splenic flexure - Descending colon - Sigmoid colon - Rectosigmoid junction |
|  |  | Size | -Longitudinal diameter (D1) (mm) *[Numeric]*  -Wall thickness (D2) (mm) *[Numeric]* |
|  | | T parameter | T2  T3  T4a  T4b (specify organ/s [free text]) |

| Lymph node metastases |  | | |
| --- | --- | --- | --- |
| Loco-regional | Yes/No  If yes:   - N1a - N1b - N1c - N2a - N2b   Site *[free text]* | | |
| Distant metastases  **according to RECIST 1.1 criteria* | Yes/No  If yes:   - M1a - M1b - M1c   Specify   1. Liver: Yes/No, Number *[Numeric]*   -If N≤2 lesion, specify for each lesion:   - 2D diameters (max, min): (mm) *[Numerico]*, (mm) *[Numeric]* - site *[liver segment]* - lesion structure *[free text]* - baseline liver structure (steatotic/non-steatotic) - relationship with vascular and biliary structures   -hepatic veins (right/middle/left; distance >1cm, <1cm, infiltration)  -hepatic veins/IVC junction (distance >1cm, <1cm, infiltration  -IVC (distance >1cm, <1cm, infiltration)  -biliary tract (distance >1cm, <1cm, infiltration)  -hepatic hilum infiltration (yes/no)  -diaphragm infiltration (yes/no)  -gallbladder infiltration (yes/no)  -colon infiltration (yes/no)  -If N>2 lesions, specify:   - number of visible lesions *[Numeric]* - 2D diameters of up to 2 target lesions* (max, min): (mm) *[Numeric]*, (mm) *[Numeric]* - structure of up to 2 target lesions *[free text]*  1. Lung: Yes/No, Number *[Numeric]*   -Specify for each of up to 2 target lesions*:   - site *[Lung lobe, Lung segment]* - 2D diameters (max, min): (mm) *[Numeric]*, (mm) *[Numeric]* - structure *[free text]*  1. Other organs (incl. skeleton): Yes/No (If yes, specify *[free text]*) 2. Non-regional lymph nodes:  - 2D diameters (max, min): (mm) *[Numeric]*, (mm) *[Numeric]* - site *[free text]* | | |
| Peritoneal carcinomatosis | Yes/No  If yes:  PCI *(link to calculator for PCI computation)* | Quadrant 0 *[Numeric]*  Quadrant 1 *[Numeric]*  Quadrant 2 *[Numeric]*  Quadrant 3 *[Numeric]*  Quadrant 4 *[Numeric]*  Quadrant 5 *[Numeric]*  Quadrant 6 *[Numeric]*  Quadrant 7 *[Numeric]*  Quadrant 8 *[Numeric]*  Proximal jejunum *[Numeric]*  Distal jejunum *[Numeric]*  Proximal ileum *[Numeric]*  Distal ileum *[Numeric]*  Total score *[Numeric]*  Specify:   - Treitz involvement (Yes/No) - Falciform ligament involvement (Yes/No) - Round ligament involvement (Yes/No) - Hepatic hilum involvement (Yes/No) - Infiltration of nearby abdominal organs   (i.e., stomach, pancreas, spleen, liver, large bowel) (Yes/No) |  |
|  | Notes | *[free text]* |  |
| Complications | Free peritoneal fluid | Yes/No |  |
|  | Bowel perforation | Yes/No  If yes: covered / uncovered |  |
|  | Bowel occlusion | Yes/No |  |
|  | Pulmonary embolism | Yes/No |  |
|  | Other | *[free text]* |  |

| T, N, M, Stage*  (TNM Classification, 8th Edition, AJCC-UICC 2017)  **web link to TNM classification* | - Tx - T0 - Tis - T1 - T2 - T3 - T4 - T4a - T4b | - Nx - N0 - N1 - N1a - N1b - N1c - N2 - N2a - N2b | •Mx  •M0  •M1  •M1a  •M1b  •M1c | - Stage 0 - Stage I - Stage IIa - Stage IIb - Stage IIc - Stage IIIa - Stage IIIb - Stage IIIc - Stage IVa - Stage IVb |
| --- | --- | --- | --- | --- |
| Incidental findings | Brain | *[free text]* | | |
|  | Neck | *[free text]* | | |
|  | Chest | *[free text]* | | |
|  | Abdomen | *[free text]* | | |
|  | Other | *[free text]* | | |

| CONCLUSIONS | *[free text]* |
| --- | --- |

**IMAGES**

| FIELD | DETAIL | ADMITTED VALUES |
| --- | --- | --- |
| Key images | Key images | *[Image]* |


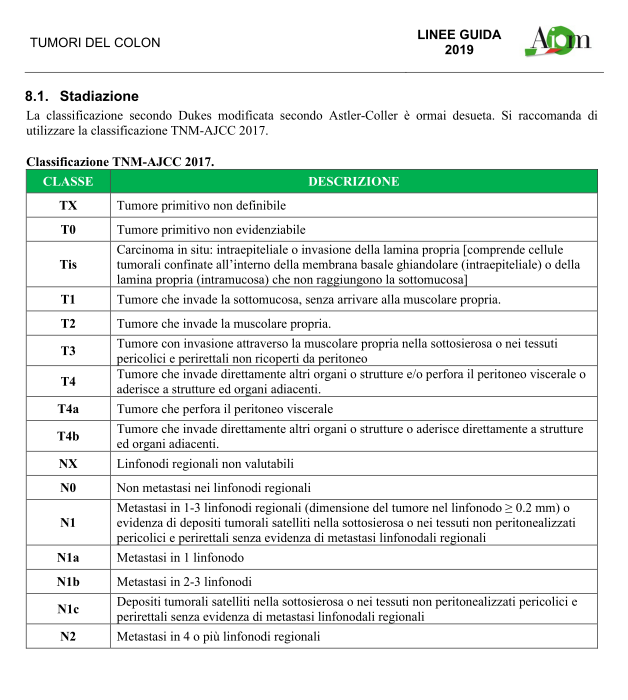


**
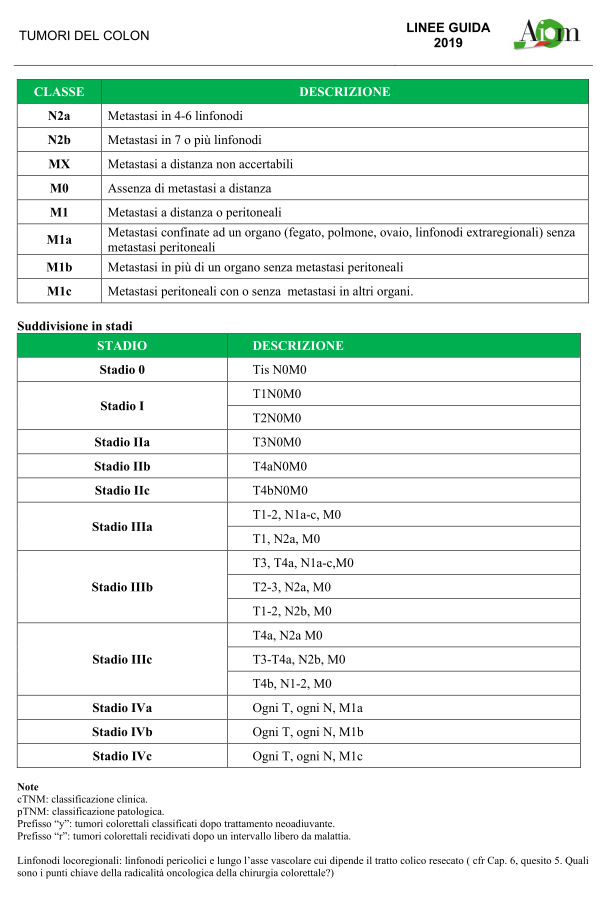
**
